# Multiple ctDNA- based biomarkers predict benefit from selective RET Inhibition in non-small cell lung cancer patients: exploratory analysis of a prospective study

**DOI:** 10.1186/s40364-025-00809-8

**Published:** 2025-07-23

**Authors:** Chang Lu, Chong-Rui Xu, Yi-Chen Zhang, E-E Ke, Yue-Li Sun, Xiao-Yan Bai, Zhi-Hong Chen, Jian Su, Yu Deng, Ting Hou, Fei Zhao, Min Li, Bin-Chao Wang, Hai-Yan Tu, Zhen Wang, Xu-Chao Zhang, Hua-Jun Chen, Jin-Ji Yang, Wen-Zhao Zhong, Qing Zhou, Yi-Long Wu

**Affiliations:** 1 Guangdong Lung Cancer Institute, Guangdong Provincial People’s Hospital (Guangdong Academy of Medical Sciences), Southern Medical University, Guangzhou, China; 2 Guangdong Provincial Key Lab of Translational Medicine in Lung Cancer, Guangdong Provincial People’s Hospital (Guangdong Academy of Medical Sciences), Southern Medical University, Guangzhou, China; 3 Burning Rock Biotech, Guangzhou, China

**Keywords:** RET, Non-small-cell lung cancer, Tyrosine kinase inhibitor, Pralsetinib, Circulating tumor DNA

## Abstract

Selective RET inhibitors such as pralsetinib have become the standard of care for patients with *RET* fusion-positive non-small cell lung cancer (NSCLC). Serial analysis of circulating tumor DNA (ctDNA) has proven effective in monitoring disease control/progression and therapeutic response in NSCLC. In this prospective study, we analyzed longitudinal ctDNA profiles (at baseline, week 8, and at progression) in Chinese patients with advanced *RET* fusion-positive NSCLC treated with pralsetinib (NCT03037385), utilizing allele frequency-based, cfDNA quantity-normalized, and methylation-based metrics. Associations between ctDNA dynamics, tumor response, and genomic alterations were assessed. A total of 21 patients were enrolled. Baseline *PIK3CA* co-mutations were associated with inferior progression-free survival (PFS; 3.0 vs. 12.4 months, *P* < 0.001). Superior PFS was observed in patients with lower baseline ctDNA levels across all metrics: allele frequency-based (HR = 0.24; 95% confidence interval [CI], 0.07–0.80; *P* = 0.012), cfDNA quantity-normalized (HR = 0.20; 95% CI, 0.05–0.71; *P* = 0.006), and methylation-based (HR = 0.09; 95% CI, 0.01–0.85; *P* = 0.010). Early ctDNA clearance at the first radiographic assessment was also associated with prolonged PFS (median PFS not reached vs. 4.8 months; *P* = 0.002) and enhanced disease control (71.4% vs. 0%). Moreover, three distinct ctDNA dynamic profiles—clearance-rebound, reduction-rebound, and sustained clearance—were associated with different progression patterns (systemic progression, new extrathoracic lesions, new intracranial/intrathoracic lesions). No evidence of histologic transformation was identified at the time of progression. *KRAS* G12R and other non-canonical alterations emerged in ctDNA-rebound samples. Molecular progression preceded radiographic progression by a mean interval of 2.2 months. These findings suggest that ctDNA-based surveillance using multiple metrics, enables early forecasting of tumor response and progression in *RET* fusion-positive NSCLC. Early ctDNA clearance and dynamic profiles provide non-invasive biomarkers for early intervention, warranting further validation with ctDNA-guided strategies.

**Supplementary Information:**

The online version contains supplementary material available at 10.1186/s40364-025-00809-8.

**To the Editor**.

Rearranged during transfection (RET) fusions are identified in 1–2% of advanced non-small cell lung cancers (NSCLC) and are targetable with potent inhibitors such as pralsetinib [1]. However, early identification of responders and resistance remains challenging, and resistance mechanisms are not yet fully understood. Liquid biopsy-based biomarkers, such as plasma circulating tumor DNA (ctDNA) provide noninvasive and longitudinal access to disease course surveillance. Previous studies have demonstrated the power of plasma cell-free DNA (cfDNA) analysis in monitoring targetable *RET* variants and ctDNA clearance after treatment initiation [2]. However, these findings require validation in studies with longer follow-up and more detailed delineation of ctDNA dynamics. Additionally, several ctDNA-derived metrics have been proposed but require further performance evaluation.

As illustrated in Fig.S1, we conducted an exploratory analysis of a prospective cohort of 21 Chinese NSCLC patients receiving pralsetinib (NCT03037385). This analysis was based on the objective response rate (ORR), the primary endpoint of the parent study, as well as progression-free survival (PFS) data from the parent study. Patient characteristics are presented in Table S1. Peripheral blood collected at baseline, week 8, at progression, was processed within 2 h to separate plasma using standardized protocols, and stored at − 80 °C. cfDNA was extracted using the QIAamp Circulating Nucleic Acid kit, quantified by Qubit, and sequenced at a depth of 20,000×. Three cfDNA indices —variant allele frequency (maxAF), cfDNA quantity-normalized mean tumor molecules (MTM/mL), and methylation-based malignancy density (MD) ratio—were calculated. The MD ratio was derived from bisulfite sequencing and machine learning-based methylation signature modeling. Full details are provided in the Supplementary Methods. Clinical outcomes were correlated with ctDNA dynamics, and genomic profiling of cfDNA facilitated the identification of potential secondary resistance mechanisms to pralsetinib. The median follow-up duration was 17.5 months (95% confidence interval [CI] 12.1–21.8 months).

## Pre-treatment ctDNA features are associated with outcomes to pralsetinib

**Fig. 1:**
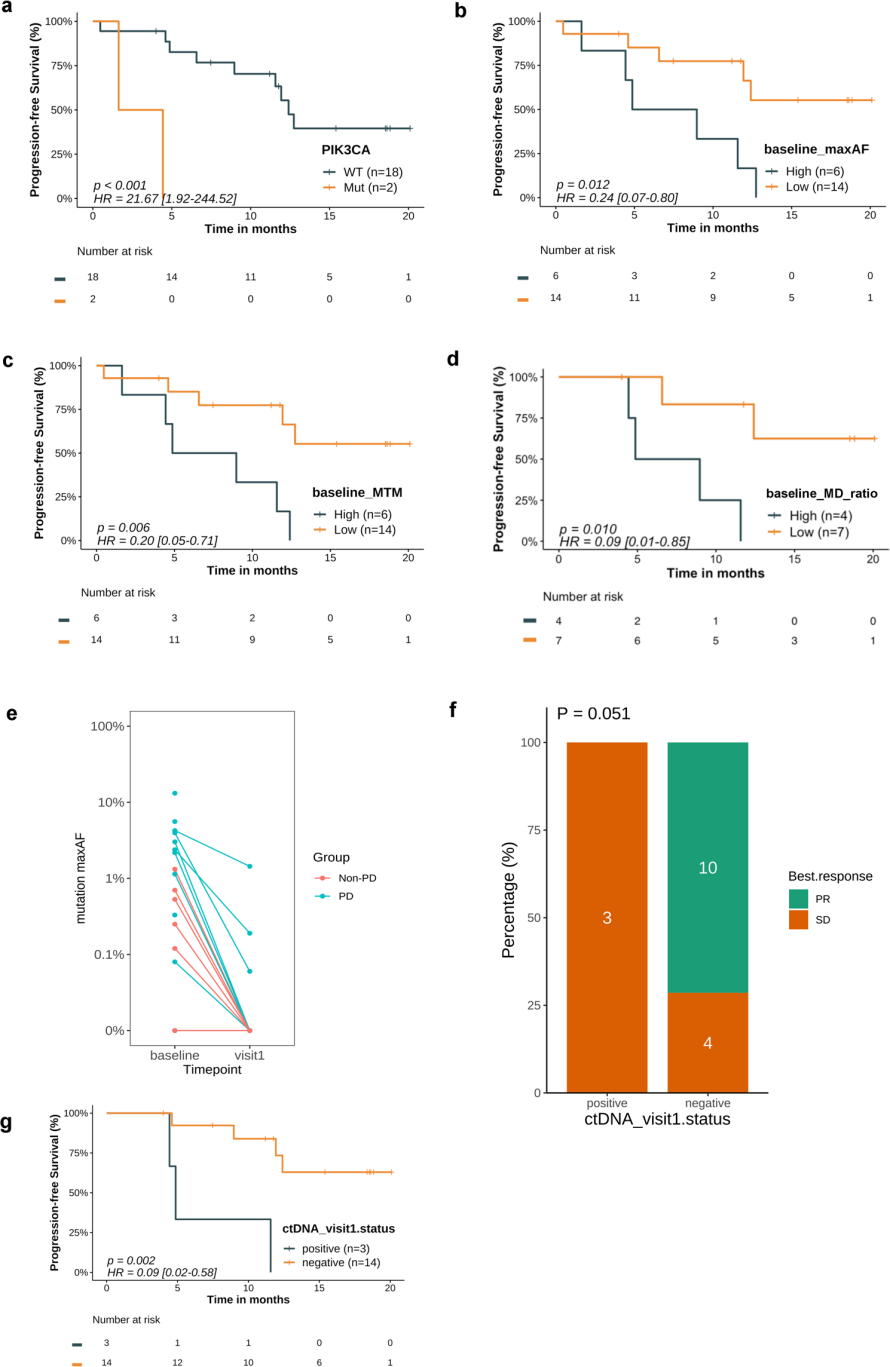
Baseline ctDNA features and early ctDNA kinetics correlate with survival and treatment response. **a**, Kaplan–Meier curves of PFS among patients with or without baseline co-occurring *PIK3CA* activating mutations at baseline are associated with poor responses to pralsetinib. **b**,** c**,** d**, Kaplan–Meier curves of PFS in the entire cohort (*N* = 21) stratified by the median value assessed by allele frequency-based ctDNA levels (**b**), cfDNA quantity-normalized ctDNA levels (**c**), and methylation-based levels (**d**). **e**, Change in ctDNA levels from baseline to the first visit (at week 8). **f**, Kaplan–Meier curves of PFS stratified by ctDNA clearance status at the first visit. **g**, Overall response groupings (bar chart) stratified by ctDNA clearance status at the first visit. Hazard ratios (HR) and 95% confidence intervals (CIs) are indicated for each analysis

Two patients harboring *PIK3CA* co-mutations (Fig.S2) (E545K and H1047L) exhibited inferior PFS compared to those without such co-mutations (median PFS: 3.0 vs. 12.4 months; *P* < 0.001; Fig. 1a). Multivariate analysis further confirmed that this association was independent of treatment lines (Fig.S3a). High pre-treatment ctDNA levels were correlated with shorter PFS across all ctDNA metrics (maxAF: HR = 0.24, *P* = 0.012; MTM/mL: HR = 0.20, *P* = 0.006; MD ratio: HR = 0.09, *P* = 0.010; Fig. 1b-d).

## Early on-treatment ctDNA dynamics forecast response to pralsetinib

All patients demonstrated a reduction in ctDNA levels by week 8 (Fig. 1e). Patients with extremely low ctDNA levels (maxAF < 0.1%) or ctDNA clearance (undetectable tumor-specific variants in cfDNA measured by both maxAF and MTM/mL) had significantly longer PFS [*P* < 0.001 (Fig.S4); *P* = 0.002 (Fig. 1f), respectively]. The association between ctDNA clearance and PFS was also independent of treatment lines (Fig.S3b). A similar, although not statistically significant, trend was observed between ctDNA clearance and a higher ORR (10/14, 71.4% vs. 0/3, 0%, Fig. 1g).

## Three distinct ctDNA dynamic patterns correlate with different disease progression outcomes

**Fig. 2:**
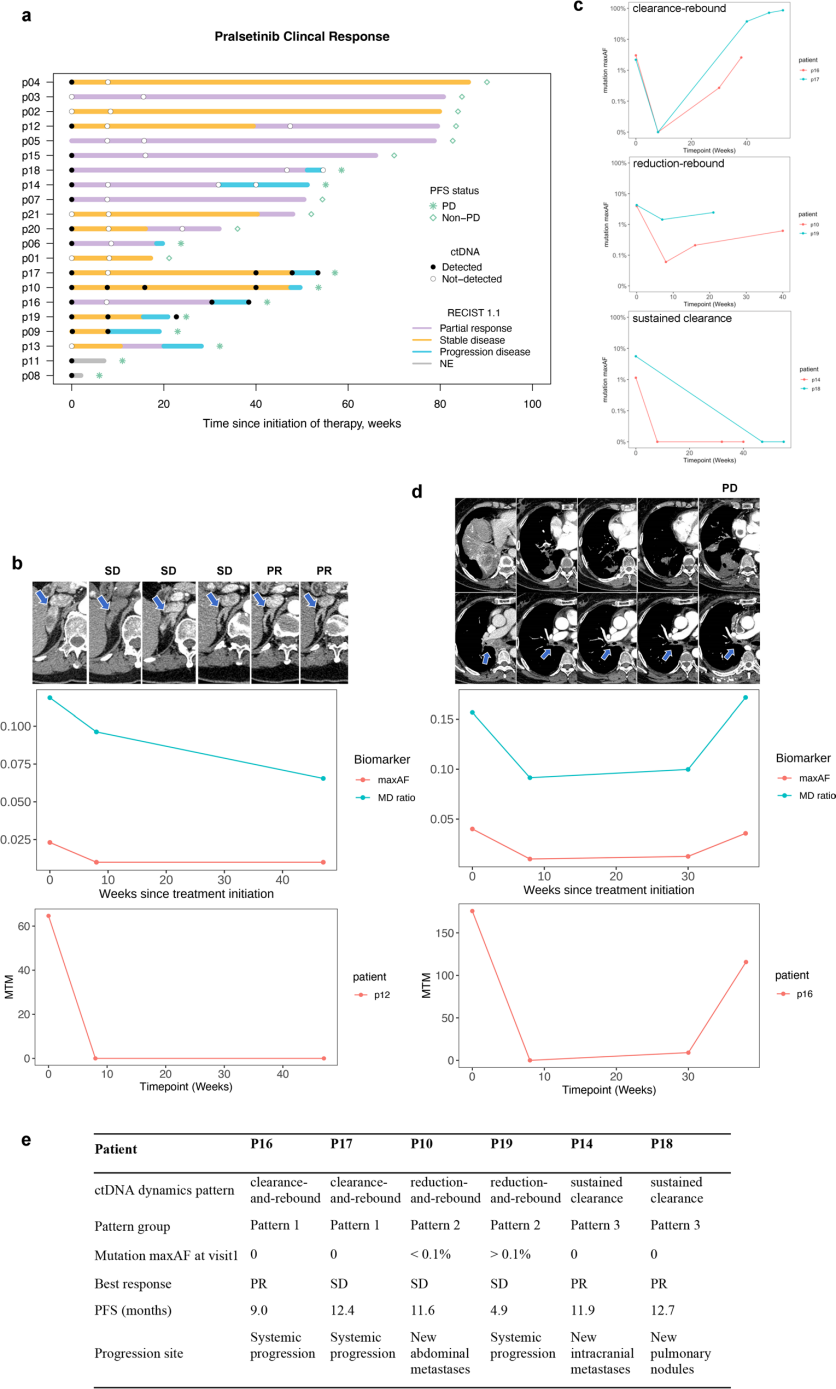
Longitudinal ctDNA monitoring. **a**, Swimmer plot depicting clinical outcomes, duration on trial and ctDNA detectability at assessed time points. Patients are ordered by ctDNA status throughout the treatment course. The total length of each bar represents duration from the first dose of pralsetinib. Colored segments indicate radiographic response at corresponding time points. **b**, Representative patient case showing ctDNA clearance followed by a delayed but durable clinical response. **c**, Definitions of ctDNA dynamic profiles: (1) Clearance-rebound (P16 and P17): ctDNA clearance at week 8 but was detected at increasing levels at PD; (2) Reduction-rebound (P10 and P19): ctDNA levels decreased but remain detectable at week 8, followed by an increase at PD; (3) Sustained clearance (P14 and P18): ctDNA clearance at week 8, remaining undetectable at PD. **d**, Representative case of a patient who progressed on pralsetinib, with serial ctDNA measurements and radiological images over the treatment course. **e**, Summary of ctDNA dynamics patterns and treatment outcomes. SD, stable disease; PR, partial response; PD, progression disease; PFS, progression-free survival

Beyond clearance, ctDNA level fluctuations also mirrored tumor burden changes. All three ctDNA metrics were correlated with RECIST measurements (Fig.S5). Clinical outcomes and ctDNA detectability at key time points are summarized in Fig. 2a and Fig.S6a. Notably, early ctDNA decline often preceded radiographic response, particularly in three patients who experienced delayed but durable response (Fig. 2a, b, Fig.S6b).

Three distinct ctDNA dynamic patterns emerged among six patients with progressive disease (PD; Fig. 2c): Clearance-rebound– ctDNA was cleared at week 8 but re-emerged at progression; Reduction-rebound– ctDNA decreased but remained detectable at week 8 and increased at progression; Sustained clearance– ctDNA was cleared at week 8 and remained undetectable at progression. ctDNA rebound was defined as an increase in maxAF of ctDNA following prior reduction or clearance.

Patients in the sustained clearance group progressed in anatomical site where ctDNA may not shed (bilateral sub-centimeter pulmonary nodules or brain-only lesions). While this may explain the absence of detectable ctDNA, other possibilities—such as intra- or inter-tumor heterogeneity, limited assay sensitivity for extremely low-frequency mutations, or undetected molecular alterations not included in our panel—cannot be excluded. Molecular progression was defined with reference to prior studies in *EGFR*-mutant NSCLC [3] and includes the following events: (1) acquisition of putative resistance mutations not present at baseline, (2) a pre-existing mutation with an allele frequency that increased by more than 10% compared with baseline, or (3) any alteration detected after ctDNA clearance (defined as undetectable ctDNA or reduction of all baseline mutations to zero at the current limit of detection of the assay, not limited to *RET* fusions). Molecular progression preceded radiologic confirmation of PD by a mean lead time of 2.2 months (Fig. 2d, Fig.S6c-e).

## Mutational landscape following resistance to pralsetinib

Resistance profiling revealed acquired an *KRAS* G12R mutation; however, no on-target *RET* alterations were identified. Additional acquired oncogenic alterations included *CSMD3* amplification, a *B2M* missense mutation and an *EPHA7* E239K mutation (Fig.S7).

In this prospective study, we integrated allele frequency, cfDNA quantity-normalized, and methylation-based ctDNA metrics to monitor treatment response and progression in *RET* fusion-positive NSCLC. We demonstrated that lower baseline ctDNA levels and early ctDNA clearance were associated with longer PFS. These findings are consistent with evidence from immunotherapy [4] and targeted therapies against *EGFR*- or *BRAF*- [5–7]and build upon a previous RET-focused study [2] by combining a broader range of ctDNA-based biomarkers and providing detailed longitudinal ctDNA dynamics in a prospective cohort. By incorporating novel metrics with traditional allele frequency-based assessments, we demonstrated strong correlation between these metrics and both survival outcomes and tumor burden. The detrimental effect of baseline *PIK3CA* co-mutation supports their role in oncogenic signalling [8]and suggests potential eligibility for combination therapies, as evidenced in prior studies combining RET and PI3K pathway inhibition [9, 10]. Few cases with acquired resistance were identified, consistent with prior evidence [11, 12].

However, several limitations should be acknowledged. First, the small sample size (*n* = 21) including two cases with *PIK3CA* mutations limited robust subgroup analyses. These findings require validation in larger, independent cohorts. Second, while putative resistance mechanisms were identified, functional validation was not performed. Third, the predictive value of the three ctDNA metrics should be confirmed in independent external cohorts receiving similar treatments. Together, these results shed light on future ctDNA-guided, real-time clinical decision-making. Future trials are warranted to validate these biomarkers and integrate them into interventional study designs.

Additional methods, full statistical analyses, and supporting figures are available in the supplemental material.

## Electronic supplementary material

Below is the link to the electronic supplementary material.

Supplementary Material 1

Supplementary Material 2

## Data Availability

The datasets used and/or analyzed during the current study are available from the corresponding author on reasonable request.
