# Supplementary material for: Multiple ctDNA- based biomarkers predict benefit from selective RET Inhibition in non-small cell lung cancer patients: exploratory analysis of a prospective study
Source: Biomark Res. 2025 Jul 23;13:98. doi: 10.1186/s40364-025-00809-8 (PMC12288196; doi:10.1186/s40364-025-00809-8)
Supplement: Supplementary file 1 — Supplementary Material 1 [file 40364_2025_809_MOESM1_ESM.docx]

Multiple ctDNA-based biomarkers predict benefit from selective RET inhibition in non-small cell lung cancer patients: exploratory analysis of a prospective study

**SUPPLEMENTARY METHODS**

**Study design and clinical endpoints**

From August 20, 2019 to September 29, 2020, we enrolled 21 Chinese patients with advanced NSCLC and harboring *RET* fusion as determined by a validated test with tumor tissue. Patients were excluded if they had previously been treated with any selective RET inhibitor. Treatment was conducted at Guangdong Provincial People’s Hospital with oral pralsetinib 400 mg administered once daily. This study was approved by the institutional ethics committee. All procedures performed in studies involving human participants were in accordance with the ethical standards of the Institutional Review Board and with the 1964 Helsinki Declaration and its later amendments or comparable ethical standards. All patients provided written informed consent for biomedical research.

Radiological tumor assessment was performed according to Response Evaluation Criteria in Solid Tumors (RECIST) version 1.1 before treatment initiation (baseline) and approximately every 8 weeks during treatment until disease progression. Progression date was defined as the date of disease progression based on RECIST (v.1.1), or the date of clinical progression if the patient discontinued pralsetinib due to clinical deterioration despite not meeting criteria for RECIST progression. Progress-free survival (PFS) was defined as time from first oral dose to the date of progression, death or last follow-up, whichever occurred first. Objective response rate (ORR) was defined as proportion of patients with complete response or partial response to pralsetinib. The clinical research data has matured and stabilized by March 4, 2022, and the trial article was published in 2023^1^. Based on this, exploratory analysis was conducted.

**Blood collection and cfDNA genomic profiling**

Longitudinal peripheral blood plasma samples were collected at baseline (within seven days before first study dose), every eight weeks following the first study dose, and at disease progression (PD), on the same schedule of imaging assessments. Genomic alterations and DNA methylation analyses based on genotyping at a high sequencing depth (20000×) were successful for 52 blood samples collected, including 20 at baseline, 26 during treatment, 6 at progressive disease (PD) evaluation. Biospecimens were transported to a designated central laboratory for molecular testing. Briefly, plasma was separated from the cell pellet within 2h of collection and aliquoted for storage at −80 °C. Cell-free DNA was extracted using a QIAamp Circulating Nucleic Acid kit (Qiagen, Hilden, Germany) and quantified with a Qubit dsDNA High Sensitivity assay kit with Qubit 2.0 ﬂuorometer (Life Technologies, Carlsbad, CA, USA).

DNA isolation, library preparation, and sequencing were performed as previously described^2^. cfDNA were hybridized with oligonucleotide baits and targeted capture was performed using a gene panel of 168 lung cancer-related genes (LungPlasma; Burning Rock Biotech, Guangzhou, China), spanning 273 kilobases of the human genome. The quality and size of fragments were assessed using a High Sensitivity DNA kit on the Bioanalyzer 2100 platform (Agilent Technologies, Santa Clara, CA, USA). Indexed samples were sequenced using a NovaSeq 6000 platform (Illumina, San Diego, CA, USA) with 2 × 150 base pair cycles at target sequencing depths of 20,000× for plasma samples. Sequencing data processing was performed using optimized bioinformatics pipelines to analyze various cancer-related somatic mutations at the DNA level including single nucleotide variants, insertions/deletions, copy number variations, and gene rearrangements.

cfDNA samples were sequenced using a capture-based bisulfite sequencing panel as described previously^3-5^. The bisulfite sequencing (BS-seq) library was prepared using the brELSA^TM^ method (Burning Rock Biotech, Guangzhou, China). Custom-designed methylation profiling RNA baits, covering 80,672 CpG sites across 1.05 Mb of the human genome, were employed for target enrichment. The enriched libraries were quantified by real-time PCR (Kapa Biosciences Wilmington, MA, USA) and sequenced on NovaSeq 6000 platform (Illumina, San Diego, CA, USA).

**ctDNA-based biomarkers quantification**

Variant allele frequencies (VAFs) was defined as the ratio of the number of variant alleles to the total allele count (sum of variant and wild-type alleles). ctDNA quantity was represented with two metrics based on somatic alterations and DNA methylation, respectively. The former was mean tumor molecules (MTM)/mL of plasma, which was determined by normalizing VAFs with cfDNA and ctDNA levels and the volume of plasma using the following formula ^6^:

$$MTM =\frac{cfDNA (ng)}{3.3 pg per hGE}\times\frac{Mean VAF}{Plasma volume(ml)}= 303 \times cfDNA (ng/mL) \times Mean VAF$$

The malignancy density (MD) ratio, representing the proportion of ctDNA in cfDNA, was quantified using a previously described machine learning-based methylation prediction model^3-5^. Briefly, cancer-specific methylation blocks (MBs) were identified by comparing the methylation profiles of tumor and tumor-adjacent normal tissue samples from an in-house cohort of lung cancer patients and normal blood samples from healthy donors using limma R package. MBs with a significant difference (Benjamini-Hochberg adjusted p-values < 0.05) were chosen. MD ratio of each patient was estimated via maximum likelihood estimation to reflect the proportion of tumor methylation signature in the plasma sample.

**Statistical analysis**

We dichotomized cohorts a priori according to log-rank test or cox regression to minimize overfitting and bias. ctDNA measurements were conducted with blinding to clinical data, and patient treatment and clinical data collection were conducted with blinding to ctDNA measurements. A statistical analysis plan was designed before data analysis. Descriptive statistics were used to summarize patients and clinical characteristics, with median and range for continuous variables and frequency and percentage for categorical variables. ORR differences between the two groups were evaluated using a Fisher’s exact test. Pearson correlation analysis was used to determines the strength of the linear relationship between two continuous variables. Survival was illustrated with the Kaplan–Meier method, and log-rank test and Cox proportional hazards regression were used to compare survival outcomes. Results were considered statistically significant if the two-sided P value was < 0.05. All statistical analyses were performed using R (R version 4.1.0, R Foundation for Statistical Computing, https:// [www.R-project.org/](http://www.R-project.org/)).

REFERENCE

1. Zhou Q, Zhao J, Chang J, et al. Efficacy and safety of pralsetinib in patients with advanced RET fusion-positive non–small cell lung cancer. *Cancer* 2023;129:3239–3251.doi:<https://doi.org/10.1002/cncr.34897>

2. Mao X, Zhang Z, Zheng X, et al. Capture-Based Targeted Ultradeep Sequencing in Paired Tissue and Plasma Samples Demonstrates Differential Subclonal ctDNA-Releasing Capability in Advanced Lung Cancer. *Journal of Thoracic Oncology* 2017;12:663–672.doi:10.1016/j.jtho.2016.11.2235

3. Li H, Ma Z-L, Li B, et al. Potential utility of longitudinal somatic mutation and methylation profiling for predicting molecular residual disease in postoperative non-small cell lung cancer patients. *Cancer Medicine* 2021;10:8377–8386.doi:<https://doi.org/10.1002/cam4.4339>

4. Liang N, Li B, Jia Z, et al. Ultrasensitive detection of circulating tumour DNA via deep methylation sequencing aided by machine learning. *Nature Biomedical Engineering* 2021;5:586–599.doi:10.1038/s41551-021-00746-5

5. Chen K, Kang G, Zhang Z, et al. Individualized dynamic methylation-based analysis of cell-free DNA in postoperative monitoring of lung cancer. *BMC Medicine* 2023;21:255.doi:10.1186/s12916-023-02954-z

6. Tin A, Aushev V, Kalashnikova E, et al. Abstract 569: Correlation of variant allele frequency and mean tumor molecules with tumor burden in patients with solid tumors. *Cancer Research* 2021;81:569–569
