# Supplementary material for: Multiple ctDNA- based biomarkers predict benefit from selective RET Inhibition in non-small cell lung cancer patients: exploratory analysis of a prospective study
Source: Biomark Res. 2025 Jul 23;13:98. doi: 10.1186/s40364-025-00809-8 (PMC12288196; doi:10.1186/s40364-025-00809-8)
Supplement: Supplementary file 2 — Supplementary Material 2 [file 40364_2025_809_MOESM2_ESM.docx]

**Supplementary Figure 1. Study design and flowchart.** **a**, Study design. **b,** Flow diagram of patient inclusion in analyses. The numbers of patients included in the study and reasons for exclusion are shown.


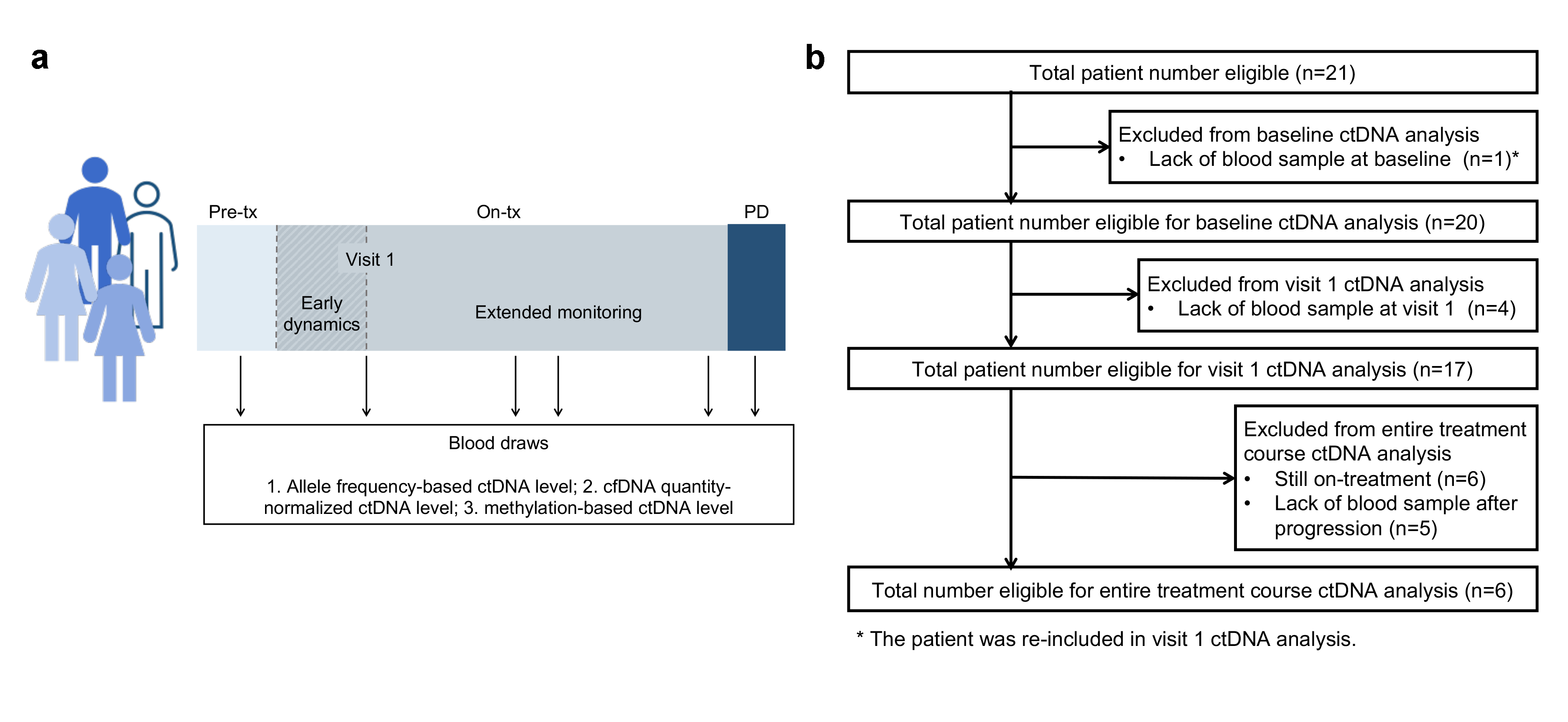


**Supplementary Table 1. Demographic and clinicopathological characteristics of the 21 patients analyzed in this study.**

| **Characteristic** | **n (%), N = 21** |
| --- | --- |
| Age, median [IQR] | 55.0 [44.0, 62.0] |
| Female | 13 (61.9) |
| Adenocarcinoma | 20 (95.2) |
| Never smoker | 14 (66.7) |
| **Tumor stage** |  |
| IVA | 5 (23.8) |
| IVB | 16 (76.2) |
| **ECOG performance status** |  |
| 1 | 21 (100.0) |
| **Prior lines of therapy** |  |
| 0 | 10 (47.6) |
| 1 | 6 (28.6) |
| 2 or more | 5 (23.8) |
| ***RET* fusion** |  |
| *CCDC6-RET* | 2 (9.5) |
| *KIF5B-RET* | 15 (71.4) |
| other | 4 (19.0) |

IQR, inter-quantile range.

**Supplementary Figure 2. Summary of gene alterations in *RET* fusion-positive NSCLC before pralsetinib.** The heatmap summarizes findings from plasma samples before treatment with pralsetinib.


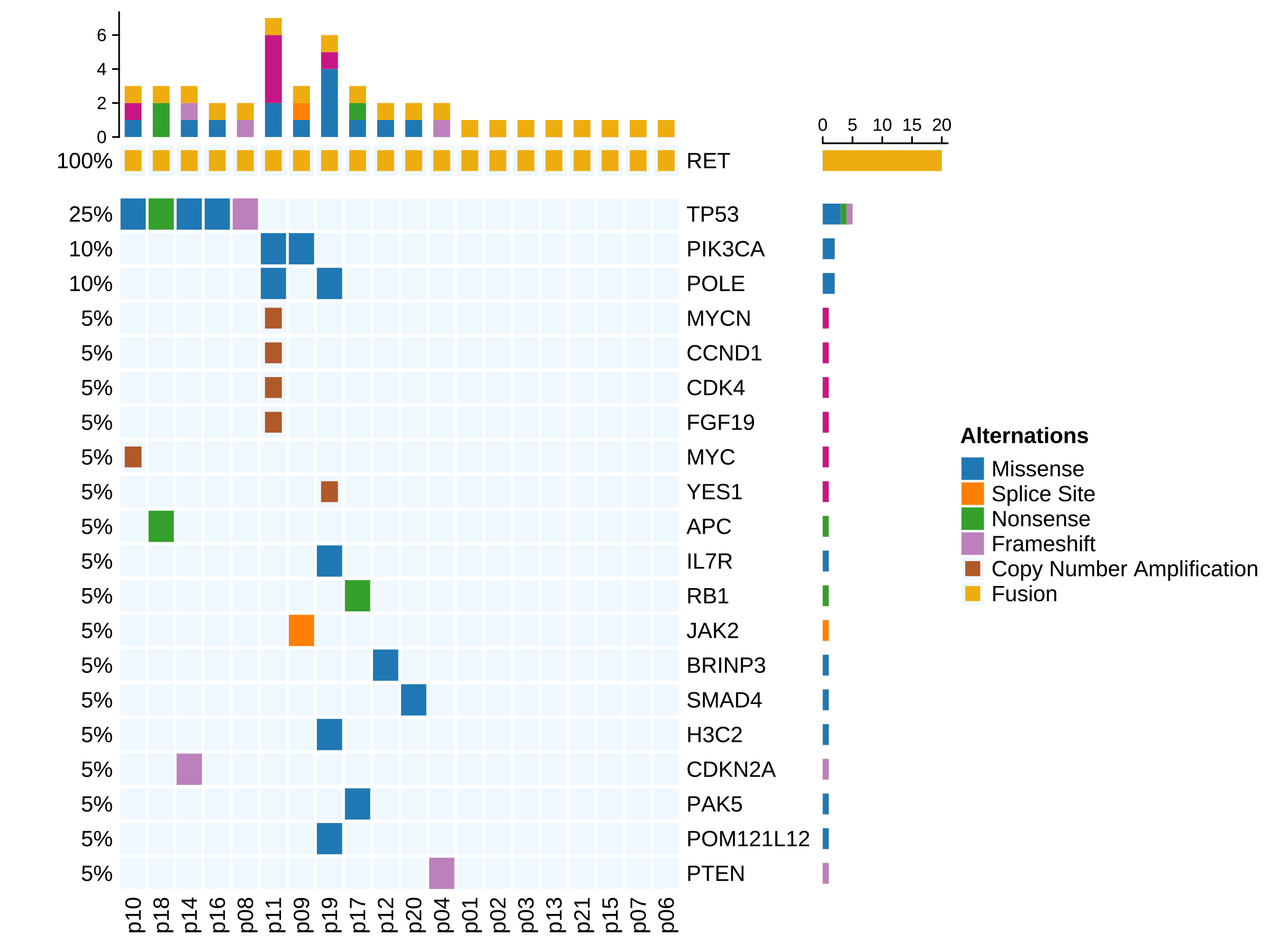


**Supplementary Figure 3.** Association of either co-occurring *PIK3CA* activating mutations at baseline (**a**) or clearance of ctDNA at visit 1 (**b**) with clinical outcomes is independent of treatment lines. Univariate and multivariate Cox regression analyses incorporating treatment line and maxAF (**c**, **d**), MTM/mL (**e**, **f**), and MD ratio (**g**, **h**) as continuous variables, with PFS as the endpoint.


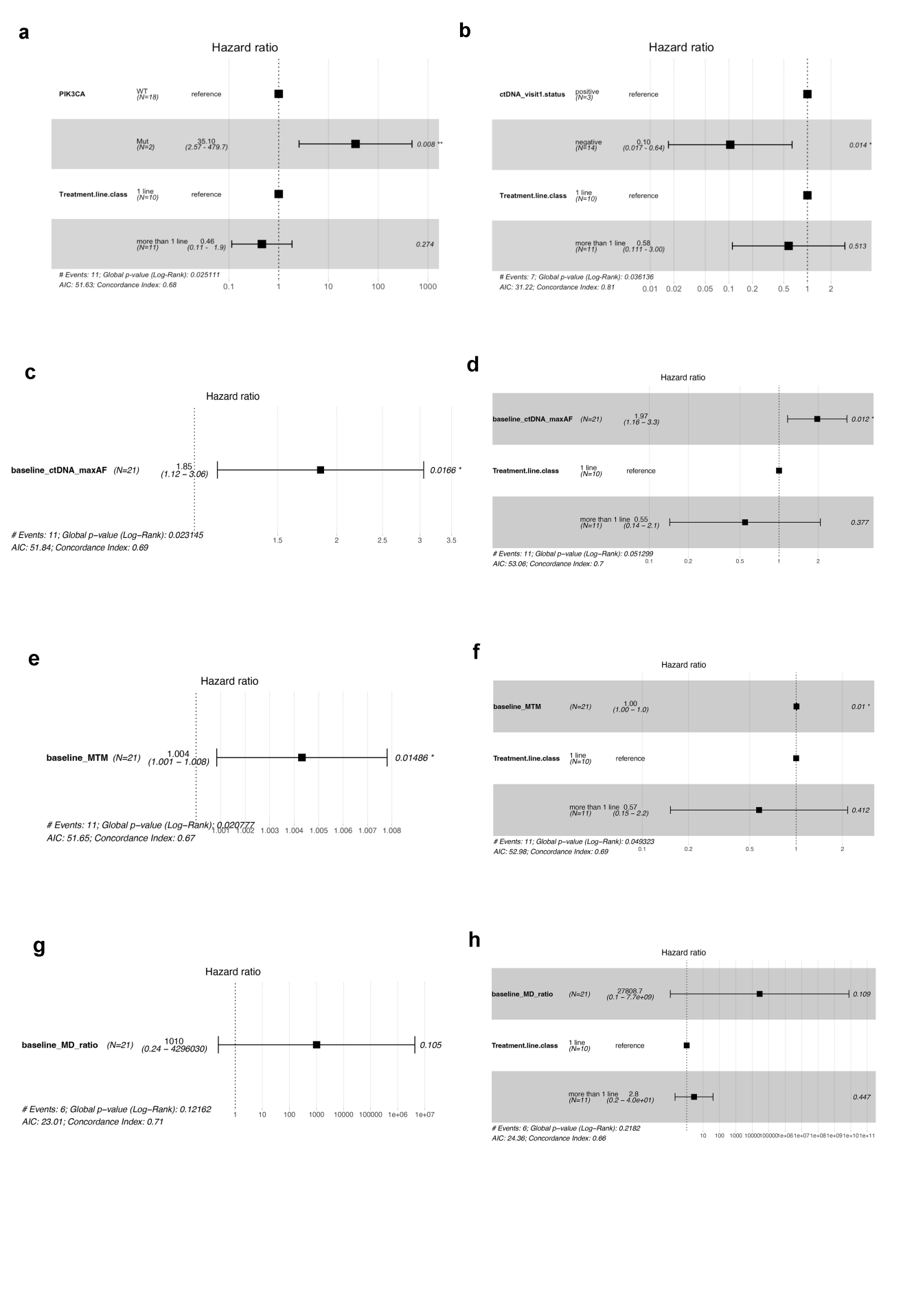


**Supplementary Figure 4.** Kaplan–Meier curves of PFS among patients with visit 1 ctDNA values stratified according to more than versus less than 0.1%.


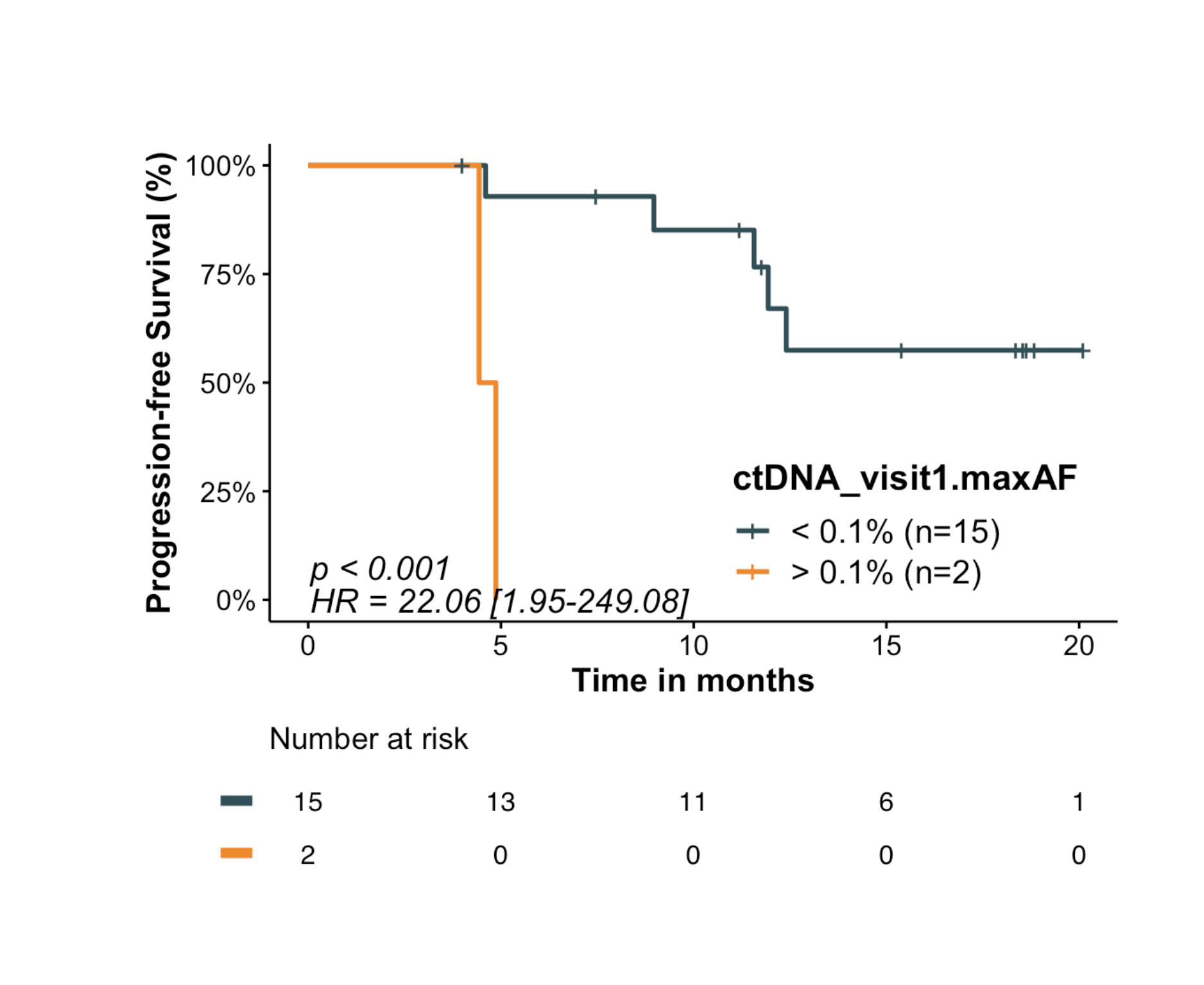


**Supplementary Figure 5. a**, Relationship between maxAF, MTM and MD ratio. Correlation between ctDNA levels and the RECIST target lesion measurements at baseline (**b**), and throughout pralsetinib treatment course (**c**).


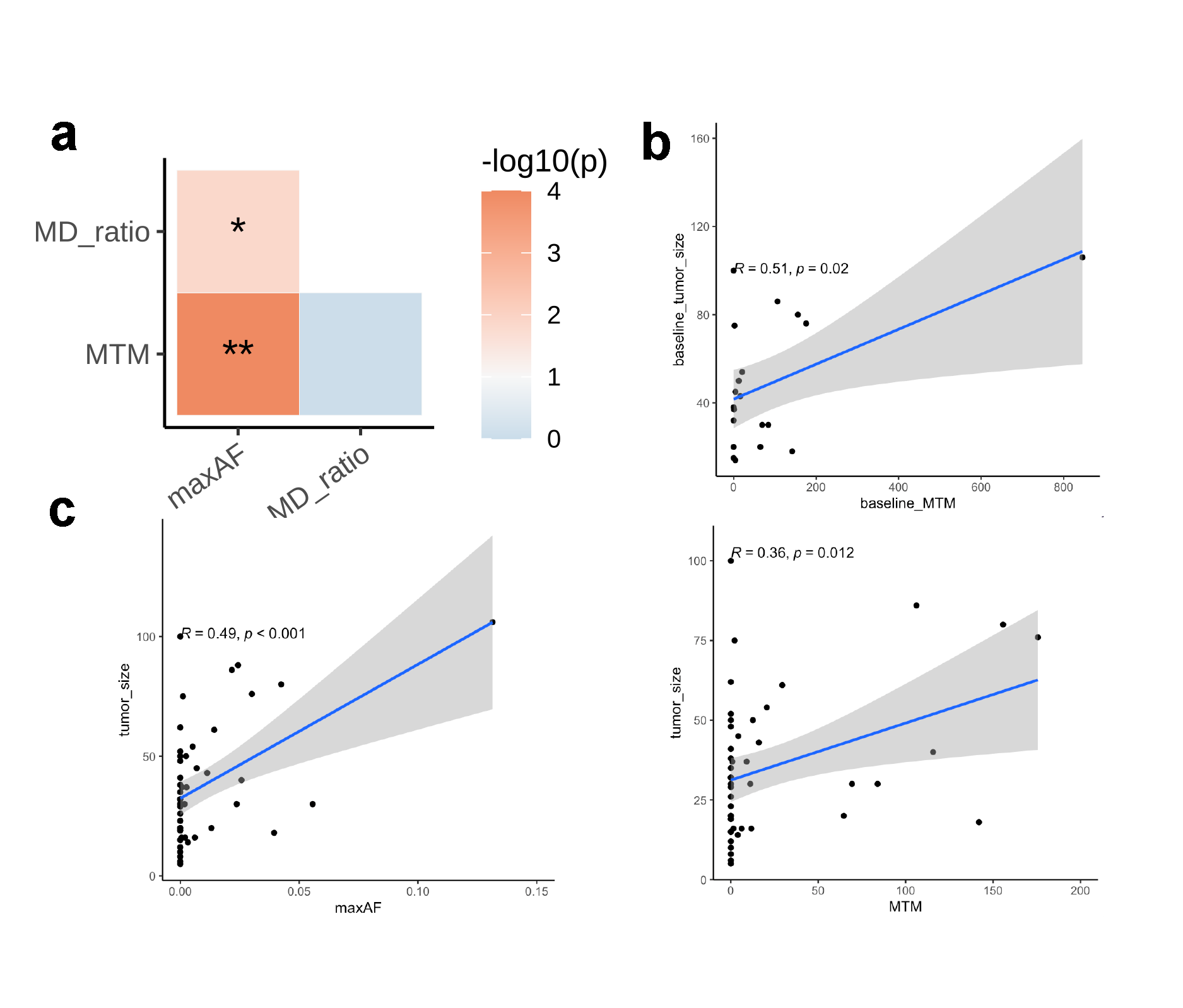


**Supplementary Figure 6. Longitudinal ctDNA monitoring.** **a**, RECIST tumor measurements during treatment grouped according to ctDNA detected at any on-treatment time. **b**, Serial ctDNA measurements during the treatment course of another patient with ctDNA clearance followed by gradual and durable clinical response. **c**, Serial ctDNA measurements during the treatment course of the remaining patients who progressed on pralsetinib.


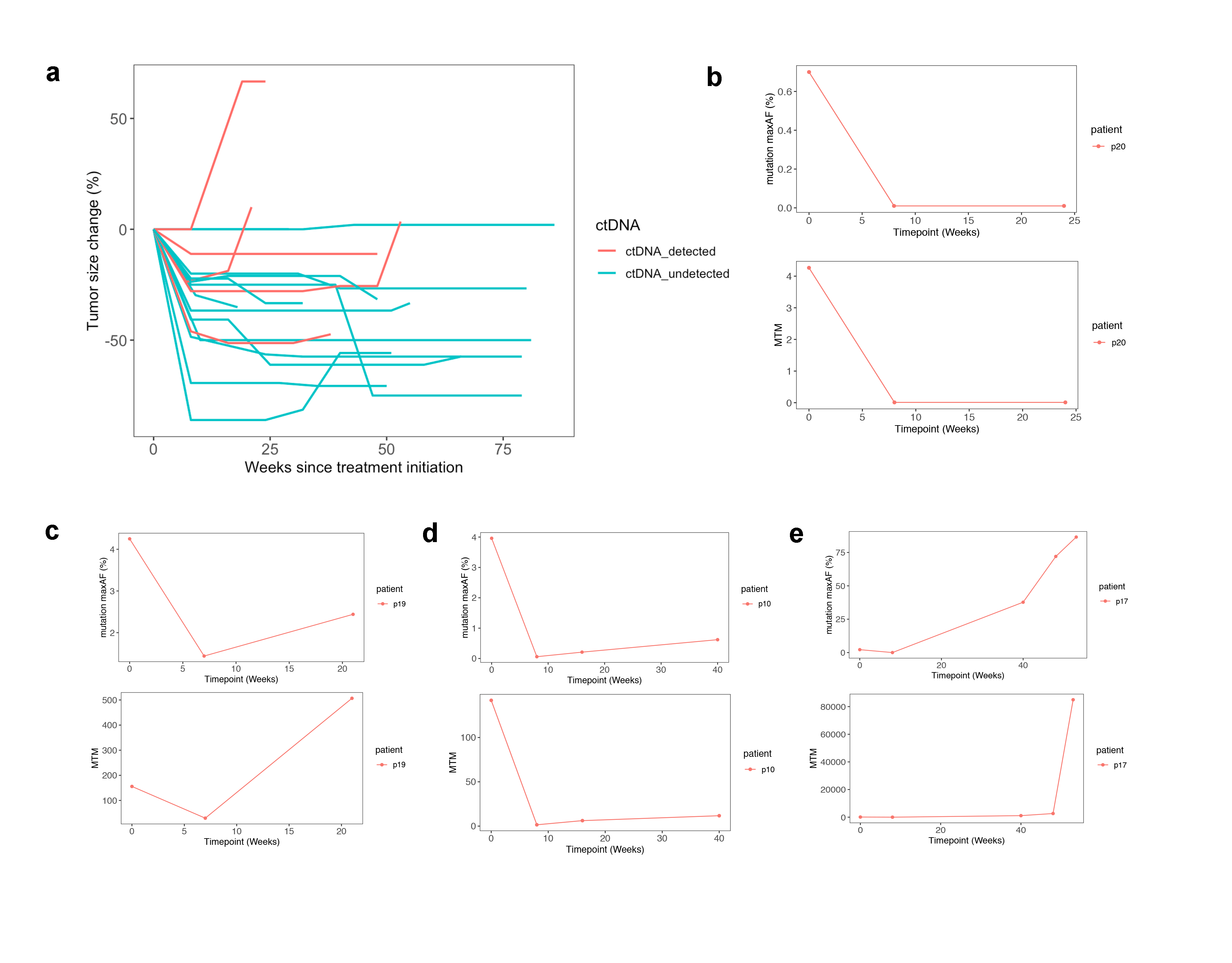


**Supplementary Figure 7. Summary of gene alterations in *RET* fusion-positive NSCLC resistant to pralsetinib.** The heatmap summarizes findings from plasma samples after treatment with pralsetinib.


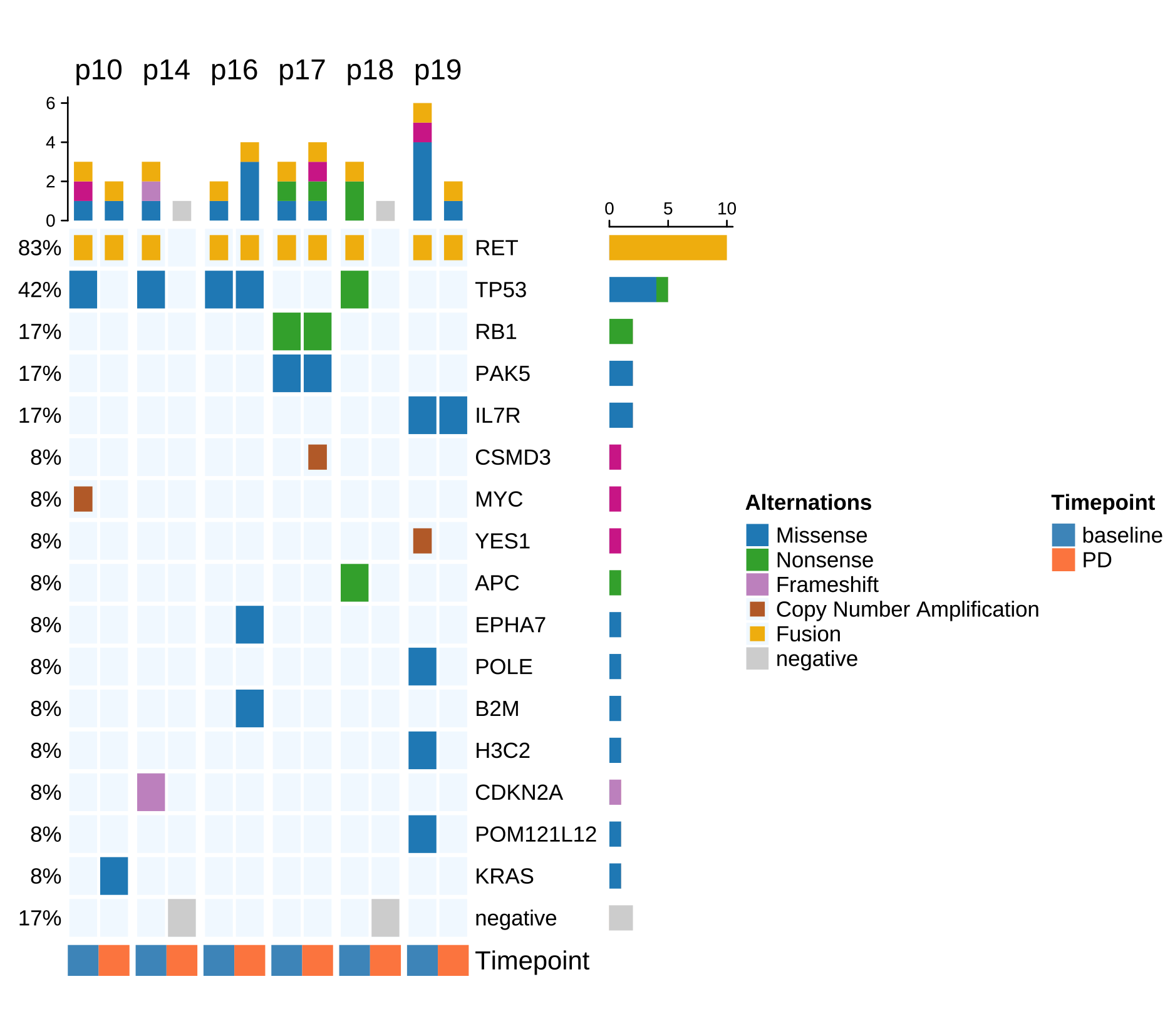


**Supplementary Figure 8. Association between MD ratio and *RET* fusion.**


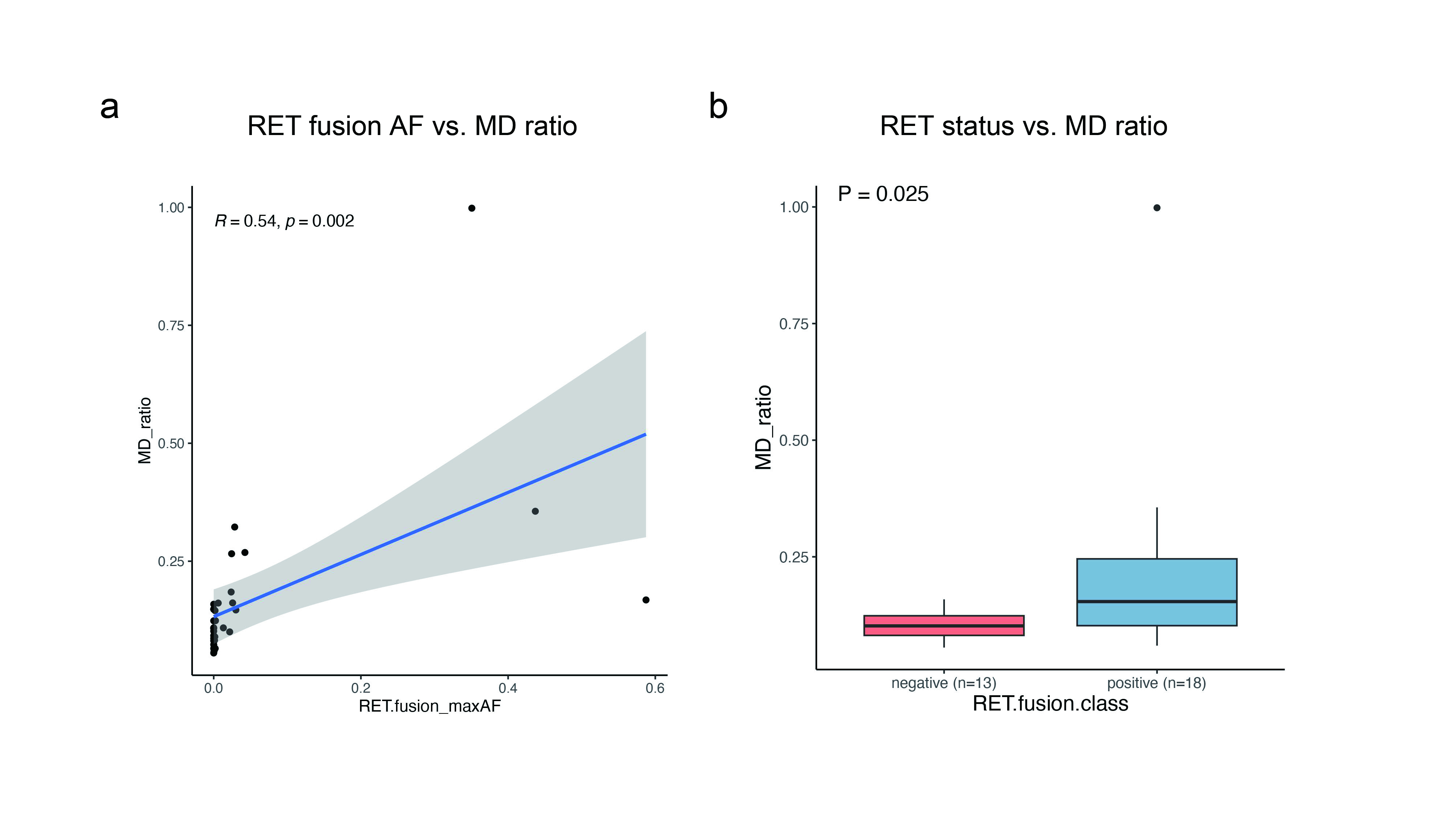


**Supplementary Table 2. the mutational profiles of the two patients (P09 and P12) with *PIK3CA* co-mutations.**

| Patient_ID | Gene | Mutation_Type | Description | AF |
| --- | --- | --- | --- | --- |
| P09 | RET | gene_fusion | KIF5B-RET | 2.37% |
| P09 | PIK3CA | missense_variant | p.H1047L | 0.11% |
| P09 | JAK2 | splice_donor_variant | c.1864+1G>T | 1.88% |
| P12 | BRINP3 | missense_variant | p.S643N | 0.81% |
| P12 | RET | gene_fusion | RET-HNRNPF | 1.31% |
| P12 | RET | gene_fusion | KIF5B-RET | 1.19% |
